# Association between Neutrophil-Lymphocyte and Platelet-Lymphocyte Ratios and Coronary Artery Calcification Score among Asymptomatic Patients: Data from a Cross-Sectional Study

**DOI:** 10.1155/2019/6513847

**Published:** 2019-03-26

**Authors:** Carlos V. Serrano, Fernando R. de Mattos, Fábio G. Pitta, Cesar H. Nomura, James de Lemos, José Antonio F. Ramires, Roberto Kalil-Filho

**Affiliations:** ^1^Heart Institute (InCor), Medical School, University of Sao Paulo, Brazil; ^2^Albert Einstein Hospital, Sao Paulo, Brazil; ^3^University of Texas Southwestern Medical Center, USA

## Abstract

**Introduction:**

Atherosclerosis is a low-grade inflammatory disease. Among markers of inflammation, importance has been given to the neutrophil-lymphocyte ratio (NLR) and platelet-lymphocyte ratio (PLR). The objective of this study was to examine the association between these hematological indices of inflammation and coronary atherosclerotic calcification in clinically asymptomatic patients.

**Methods:**

This study had clinical and laboratorial data collected from consecutive asymptomatic patients that underwent computed tomography coronary artery calcium (CAC) scoring. Risk factors, NLR, and PLR were evaluated at different categories of CAC scoring. Statistical tests included chi-square, linear regression, and logistic regression. Patients (*N* = 247; age 60.4 ± 8.0 years and 60.7% men) were allocated into four categories according to the CAC score.

**Results:**

Respective age, sex (male), NLR, and PLR distribution within groups were as follows: CAC = 0 (*n* = 98; 52.5 ± 13.6 years, 55%, 2.0 ± 1.0, and 121.5 ± 41.5), CAC 1-100 (*N* = 64; 61.3 ± 11.0 years, 60%, 2.2 ± 1.2, and 125.6 ± 45.6), CAC 101-400 (*N* = 37; 64.2 ± 11.6 years, 67%, 2.6 ± 1.3, and 125.4 ± 55.9), and CAC > 400 (*N* = 48; 69.3 ± 11.1 years, 66%, 3.3 ± 2.0, and 430.1 ± 1787.4). The association between risk factors and CAC score was assessed. Hypertension status and smoking status were similar within groups, while the presence of diabetes (*P* = 0.02) and older age (*P* ≤ 0.001) was more prevalent in the CAC > 400 group. LDL cholesterol was greater in the higher CAC score groups (*P* = 0.002). Multivariate logistic regression of the quartile analysis showed that age and NLR were independently associated with CAC > 100 (OR (CI), *P* value): 2.06 (1.55-2.73, *P* = 0.00001) and 1.82 (1.33-2.49, *P* = 0.0002), respectively.

**Conclusion:**

Within asymptomatic patients, NLR provides additional risk stratification, as an independent association between NLR extent and CAD extent was identified. Moreover, PLR was not an inflammation marker for CAD severity.

## 1. Introduction

Atherosclerotic coronary artery disease (CAD) has long been shown to represent a chronic low-grade subclinical systemic inflammatory disease. Numerous pathways and markers have been studied in order to detect the presence and evolution of this disease [1, 2]. Among markers of inflammation involved in atherosclerosis, increasing importance has been given to circulating blood components, such as subtype leukocyte and platelet counts, and future cardiovascular events in healthy subjects [3, 4]. Recent research has revealed a significant association between cardiovascular risk and the neutrophil-lymphocyte ratio (NLR) [5, 6] and platelet-lymphocyte ratio (PLR) [7, 8].

A low blood lymphocyte count has been shown to be related with worse cardiovascular consequences in patients with CAD [9, 10] and chronic heart failure [11]. In cases of sustained inflammation, lymphocyte counts decrease due to increased lymphocyte apoptosis. Lymphocytes represent a more convenient immune response, while neutrophils cause a destructive inflammatory reaction [12]. Also, ongoing inflammatory conditions lead to increased proliferation in megakaryocytic series and relative thrombocytosis. Previous studies have demonstrated an association between altered platelet parameters and major adverse cardiovascular outcomes in patients with CAD [13] and also in healthy adults [14, 15]. PLR is a new prognostic marker that integrates the risk prediction of these 2 parameters into 1. It gives an idea about both the aggregation and inflammation pathways, and it may be more valuable than either platelet or lymphocyte count alone in the prediction of coronary atherosclerotic burden [16]. Therefore, these markers offer an effective, simple, and relatively cheap tool for the diagnosis and prognosis of CAD events, even in the asymptomatic individuals.

Most of the current studies that investigated associations between inflammatory biomarkers and CAC score used novel markers that might not be readily available clinically in general hospital settings and were conducted in symptomatic subjects. Data with classic inflammatory markers derived from complete blood count such as red blood cell indices, white blood cell indices, and platelet counts are still conflicting and sparse in asymptomatic patients. Whether various blood cell counts and ratios can reflect the presence and/or extent of CAC in this population with nonmanifested CAD is still uncertain.

As a subclinical measure of cardiovascular disease, CAC scoring is known to predict cardiac events and has been a valuable tool for CAD stratification of low-intermediate-risk patients [17, 18]. Associations between inflammatory markers and the amount of CAC have been reported for the general population [19]; however, few studies have looked at the association between inflammation and the degree of CAC [20].

The association of these hematological indices of inflammation and coronary calcification load has been slightly investigated.

This investigation was based on the hypothesis that among patients with nonmanifested CAD, simple hematological indices of inflammation, such as NLR and PLR, can be associated with coronary atherosclerotic burden. In spite of the vital roles played by both blood markers of inflammation and calcification in coronary atherosclerosis, no other study has documented the relationship between NLR and PLR and CAC score in patients with nonmanifested CAD.

Thus, the objective of this study was to assess the association between NLR and PLR and the extent of CAC in clinically asymptomatic patients. Blood sample collection concomitant to coronary calcium scoring was determined for this purpose.

## 2. Materials and Methods

This single-center and cross-sectional study had clinical and laboratorial information collected retrospectively from the cardiovascular imaging databank of the Heart Institute (InCor). This study was designed and managed according to the Declaration of Helsinki.

### 2.1. Study Population

Consecutive asymptomatic and stable patients, clinically considered as having low-intermediate risk for CAD events, underwent risk stratification by means of blood sample collection and concomitant cardiac computed tomography coronary artery calcium scoring.

The patients who had a leukocyte count out of the normal range (4000-12000 cells/*μ*L), acute infection, autoimmunological diseases, and any other disease (such as urinary system infection or cholecystitis) that could affect leukocyte count were excluded.

### 2.2. Cardiovascular Risk Factors

In terms of risk factors for CAD, age, sex, smoking, history of myocardial infarction, type 2 diabetes mellitus (T2DM), hypertension, and dyslipidemia were explored.

T2DM was defined in patients with a HbA1c level of ≥6.5% and a fasting plasma glucose level of ≥126 mg/dL. Hypertension was characterized by systolic pressure ≥ 140 mmHg or diastolic pressure ≥ 90 mmHg in adults (>18 years of age). These measurements had to be done in two different days and using calibrated blood pressure tools. Dyslipidemia was defined in patients with a fasting total cholesterol serum level of ≥240 mg/dL, triglyceride serum level of ≥200 mg/dL, low-density lipoprotein (LDL) cholesterol ≥ 160 mg/dL, and/or high-density lipoprotein (HDL) cholesterol < 40 mg/dL and in patients receiving or not receiving medical therapy for dyslipidemia [21].

### 2.3. Coronary Calcium Scoring

All patients underwent non-contrast-enhanced computed tomography (CT) to detect the CAC score. CT began from the carina extending to the subdiaphragmatic level (Aquilion™ 64 CFX from Toshiba America Medical Systems Inc., Tochigi, Japan). In CT, calcified coronary artery plaques were explored visually on cardiac sections. CAC score was determined using preexisting software in the device (SURECardio, Toshiba Medical Systems, Otawara, Japan; Agatston scoring method).

The total CAC score was the sum of calcium levels calculated in the left main coronary artery, in the left anterior descending coronary artery, in the circumflex coronary artery, and in the right coronary artery traces. Collected data were evaluated using the percentiles predefined according to age and sex [22]. Patients were categorized into four groups based on their CAC extent: absent (CAC = 0), mild (CAC 1-100), moderate (CAC score 101-400), and severe (CAC score > 400).

### 2.4. Blood Cell Counts

Different leukocyte subtypes and platelet counts were obtained using an automated hematology analyzer (Abbott Cell Dyn 3700; Abbott Laboratory, Abbott Park, Illinois, USA). NLR and PLR were calculated by dividing absolute neutrophil and platelet counts, respectively, by the absolute lymphocyte count. CT was performed in a short time (5-7 days) after blood cell count measurement.

### 2.5. Statistical Analysis

Patients were divided into four different categories based on their CAC score. Descriptive statistics for studied variables are presented as mean (standard deviation (SD)) for normally distributed variables, median (interquartile range (IQR)) for nonnormally distributed variables, and frequency (percentage) of categorical variables. Spearman correlation was performed between study variables and CAC score. Analysis of variance (ANOVA) and Student's *t*-test were used to identify differences in means between CAC categories. A post hoc analysis was applied to investigate in more detail the experimental data after the one-way analysis of variance (ANOVA) findings. Tukey's multiple comparisons test was used to figure out which groups in the sample differ. The “Honest Significant Difference” was applied—a number that represents the distance between groups, to compare every mean with every other mean. Kruskal-Wallis *H* test and Wilcox-Mann-Whitney *U* test were used to examine differences in medians between CAC categories. *χ*^2^ analysis was used to identify significant heterogeneity in the frequencies. Univariable and multivariable analyses were performed with binary logistic regression and linear regression. Logarithmic transformation of the CAC score was used for linear regression test. Each ratio was adjusted for the cardiovascular risk factors in multivariable analysis. All statistical tests were performed with SAS/STAT® software. A two-tailed *P* value < 0.05 was considered statistically significant [23].

## 3. Results

Consecutive 247 patients (aging 60.4 ± 8.0 years and 60.7% men), who underwent clinical evaluation, leukocyte counting, and Agatston CAC scoring during the study period, were included for the hematologic indices/CAC analysis.

As seen in Table 1, most (39.7%) had absent CAC, followed by 25.9% with mild CAC, 15.0% with moderate CAC, and 19.4% with severe CAC. The mean age of patients in the absent CAC group was lower than that of patients with CAC (overall *P* < 0.001). However, only patients with severe CAC were significantly older than patients with absent CAC (*P* = 0.008), but not patients with mild CAC (*P* = 0.06) or moderate CAC (*P* = 0.07). There were fewer men among those with absent CAC, compared to those with any extent of CAC.

Overall, hematological tests were within the normal limit according to laboratory references. There was no statistically significant difference in total leukocyte counts, relative neutrophil counts, absolute neutrophil counts, relative lymphocyte counts, absolute lymphocyte counts, or platelet counts among the CAC categories.

**Figure 1:**
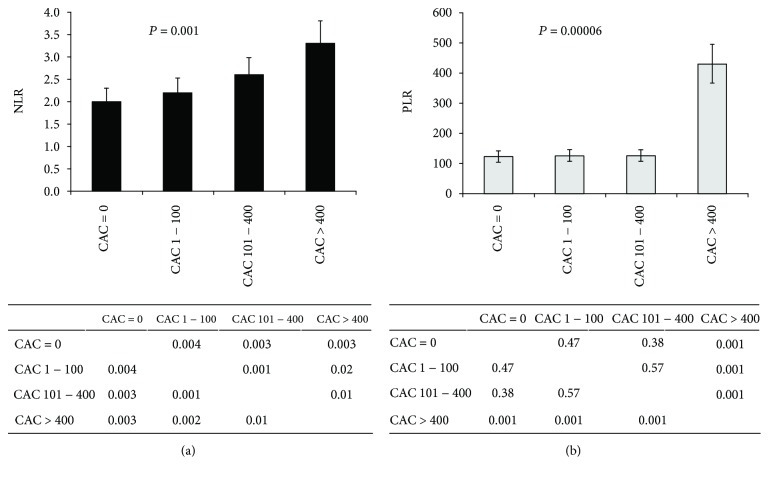
Association between the (a) neutrophil-lymphocyte ratio (NLR) and (b) platelet-lymphocyte ratio (PLR) and different levels of coronary artery calcification (CAC) in clinically asymptomatic patients. ANOVA tests revealed significant overall differences between CAC groups for both NLR and PLR. Post hoc analysis showed that for NLR, all CAC groups were significantly different from each other. CAC = 0, CAC 1-100, and CAC 101-400 groups present similar PLR levels; only CAC > 400 patients markedly diverged from the other CAC patients.

Figure 1 shows the unadjusted association between NLR and PLR and different levels of CAC in clinically asymptomatic patients. Interestingly, patients with higher CAC levels presented markedly higher NLR and PLR.

**Figure 2:**
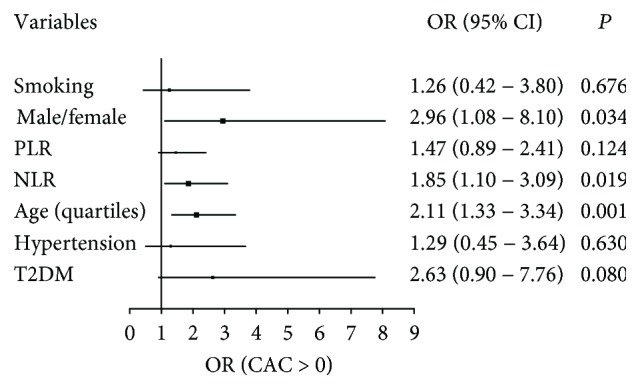
Multivariate logistic regression of asymptomatic patients for the presence of coronary artery calcification (CAC > 0). Gender (male/female), age (decades), and NLR are independently associated with CAC > 0. NLR: neutrophil-lymphocyte ratio; PLR: platelet-lymphocyte ratio; T2DM: type 2 diabetes mellitus; OR: odds ratio.

As seen in Figure 2, multivariate logistic regression analysis showed that gender, age, and NLR are independently associated with coronary calcification (CAC > 0). The addition of smoking status, T2DM, and hypertension in multivariate regression analysis did not confer difference in results. In this analysis, among asymptomatic and clinically stable patients, there is a marked positive connotation of the inflammation marker NLR, and not PLR, with the CAC extent.

## 4. Discussion

It is well understood that increases in markers of inflammation as well as the presence of coronary calcium enhance the probability of adverse short- and long-term cardiovascular events [24, 25]. In this perspective, the current study was elaborated on the hypothesis that among asymptomatic patients with no history of manifested CAD, simple hematological indices of inflammation, such as NLR and PLR, can evaluate coronary atherosclerotic burden.

The role of calcification in CAD is gaining importance, both in research studies and in clinical application. Current investigations have shown that plaque calcification has a progression that is closely related to the level of vascular inflammation [26]. In this context, we undertook an update on the interface between inflammation and coronary calcification, focusing on clinical patient risk stratification. As the total amount of calcium in the coronary arteries is increased, risk of future coronary heart disease enhances [27].

Atherosclerosis is a complex systemic disorder where coronary risk factors, such as hypertension, dyslipidemia, T2DM, and smoking, have important pathophysiological roles [28, 29]. Current reports have demonstrated that the initiation of atherosclerosis involves a dynamic inflammatory activity, and not just an inert vascular injury instigated by blood lipoproteins and other elements on the arterial wall [30, 31]. Also, white blood cells have a key participation in this inflammatory course [32, 33]. Contemporary studies have revealed that neutrophils play an important role in the various stages of atherosclerosis, from the progress to thedestabilization of plaques [34, 35]. In support of this reasoning, this study demonstrated, after logistic multiple regression analysis, that NLR is independently associated with CAC > 0.

Selected investigations registered that dyslipidemia triggers an increase in neutrophil levels by boosting the production of these cells in the bone marrow as well as diminishing their blood clearance [36, 37]. However, our results did not show any differences among the CAC groups regarding dyslipidemia. Overall, the mean NLR was 2.5 ± 0.9 in the patients with LDL cholesterol > 130 mg/dL and was 2.3 ± 0.6 in those with LDL cholesterol ≤ 130 mg/dL. This finding suggests that the differences in NLR regarding CAC scores were independent of cholesterol levels.

As inflammation markers, NLR and PLR are increasingly being referred in clinical cardiovascular practice since these ratios can reflect an acute episode of inflammation (increase in neutrophil and platelets) and acute physiological stress (decrease in lymphocyte) [38, 39]. In acute coronary syndromes, lymphopenia can be observed together with the increase in neutrophils [40, 41]. Leukocytes and subtypes can predict cardiovascular events as well as an indicator of inflammation followed by a myocardial infarction [42]. Kirtane et al. [43] showed that the increase in neutrophil level was associated with extent and short-term prognosis of myocardial damage in acute coronary syndromes. Thomson et al. [44] reported that the lymphopenia observed in acute coronary syndromes was associated with stress-induced cortisol release and that this was one of the early findings. It is essential to register that in this investigation, the participants included were patients who had no acute coronary syndrome, neither findings to coronary angiography indication nor suspicious laboratory results. Being stable patients, this study proposes a novel role for these ratios in the setting of the investigation of CAD in asymptomatic patients.

Few investigations have emphasized NLR and PLR to be independent predictors of cardiac events, comprising mortality, in patients with CAD [45, 46]. Elevated NLR has been shown to be associated with the decrease in survival after revascularization procedures [47, 48]. In the study investigating the progression of coronary atherosclerosis, Kalay et al. [49] reported NLR as an important indicator. In this study, NLR and PLR were not compared with cardiac events as a predictor. But according to the findings, we can assume that coronary artery plaque accumulation (determined by CAC scoring), which has a potential for cardiac events, was higher within patients with superior NLRs, independent of the presence of risk factors.

Interestingly, this study revealed significant overall differences between CAC groups for both NLR and PLR. Particularly, NLR presented a direct independent association with the levels of CAC. However, only CAC > 400 patients markedly diverged from the other CAC patients regarding PLR; CAC = 0, CAC 1-100, and CAC 101-400 groups presented similar PLR levels.

The role of calcification in CAD is gaining importance, both in research studies and in clinical application. Recent analyses have shown that plaque calcification has a dynamic progression that is narrowly related to the magnitude of vascular inflammation [50]. In this context, we undertook an update on the interaction between inflammation and coronary calcification, focusing on clinical implications such as patient risk stratification [51].

The relationship between CAC and other inflammatory markers has also been extensively studied. Most investigations demonstrated positive but weak association between these biological markers and the presence or extent of CAC. C-reactive protein (CRP) is one of the most studied markers. It has been shown that, in asymptomatic subjects without apparent CAD, high CRP was associated with the presence of CAC [52, 53]. In addition, the value of CRP was also shown to correlate with the value of CAC [54].

Since a positive CAC score by computed tomography has a strong association with total coronary atherosclerosis load and the risk of cardiovascular events, we compared multiple variables regarding the absence (CAC score = 0) and presence (CAC score > 0) of coronary calcification. Various studies have shown that asymptomatic patients with a CAC score of zero have a low risk of cardiovascular events or all-cause mortality in the medium term and long term [55]. Importantly, NLR was independently associated with CAC > 0, along with gender (male/female) and age (decades).

In summary, we have demonstrated a significant association between NLR, and not PLR, and CAC in asymptomatic patients suspected for CAD. These findings are in agreement with most of the other current studies in literature, regarding a link between inflammatory markers and CAD. This consistency might possibly be from the robustness of NRL in detecting the chronic low level of inflammation in atherosclerotic CAD. Even more, these findings suggest that NLR can be a reliable marker of CAC in a clinical setting.

### 4.1. Clinical Relevance

We call to attention that, to our knowledge, no other clinical investigation has shown such noticeable association between coronary calcium scoring, detected by cardiac CT, and subtype leucocyte ratios, which are readily available in clinical practice. The original appreciation of this investigation is the simultaneous assessment of both CAC and inflammation markers in asymptomatic and stable patients. In previous studies, these features were always evaluated separately and within high-risk patients.

### 4.2. Study Limitations

The present study mostly comprised stable patients who underwent CT and was designed as a single-center cross-sectional investigation. In addition, CRP, a well-accepted marker of acute-phase inflammatory response, was not determined in this study for terms of comparison to the hematologic indices studied, mainly due to the reason that it was not the focus of this study.

As the only observational study design that assures the temporal relationship between exposure and outcome, epidemiological/prospective studies provide the best level of evidence for any potential role of hematological indices of inflammation, as well as circulating CRP, in onset of coronary calcification. Thus, additional studies are needed to verify the results of this study.

## Figures and Tables

**Table 1:** Baseline characteristics of 247 clinically asymptomatic patients according to levels of coronary artery calcification score.

	CAC = 0 (*N* = 98, 39.7%)	CAC 1-100 (*N* = 64, 25.9%)	CAC 101-400 (*N* = 37, 15.0%)	CAC > 400 (*N* = 48, 19.4%)	*P*
Clinical data					
Age (years)	52.5 ± 13.6	61.3 ± 11.0	64.2 ± 11.6	69.3 ± 11.1	≤0.001
Men (%)	55	60	67	66	<0.001
Current or past smoker (%)	20.8	21.5	22.1	23.1	0.06
History of MI (%)	18.1	21.7	22.7	28.9	<0.001
History of hypertension (%)	35.9	37.8	36.9	36.9	0.12
History of T2DM (%)	25.8	27.1	28.2	33.7	0.02
Laboratorial data					
TC	180.1 ± 24.6	185.2 ± 23.6	186.3 ± 21.3	188.1 ± 21.5	<0.007
LDL-C	101 ± 23.0	102 ± 21.6	111 ± 19.911	116 ± 23.7	0.002
HDL-C	42.4 ± 10.5	42.6 ± 10.6	41.8 ± 10.7	42.1 ± 9.8	0.78
TG	145.5 ± 24.7	144.8 ± 21.1	146.3 ± 21.2	145.6 ± 19.9	0.13
WBC	5.96 ± 1.59	6.86 ± 2.39	4.95 ± 1.70	5.70 ± 2.39	0.08
Neutrophil	4.00 ± 4.7	3.35 ± 4.3	3.91 ± 4.7	4.95 ± 5.7	0.06
Lymphocyte	2.01 ± 5.0	1.52 ± 5.0	1.51 ± 5.0	1.50 ± 4.7	0.70
Platelet	243.8 ± 24.7	191.1 ± 34.1	189.4 ± 24.7	645.1 ± 12.1	0.06
NLR	2.0 ± 1.0	2.2 ± 1.2	2.6 ± 1.3	3.3 ± 2.0	0.001
PLR	121.5 ± 41.5	125.6 ± 45.6	125.4 ± 55.9	430.1 ± 1787.5	0.00006

CAC: coronary artery calcification score; MI: myocardial infarction; T2DM: type 2 diabetes mellitus; TC: total cholesterol; LDL-C: LDL cholesterol; HDL-C: HDL cholesterol; TG: triglycerides; WBC: white blood cell; NLR: neutrophil-lymphocyte ratio; PLR: platelet-lymphocyte ratio. Age expressed in mean ± SD; lipids in mg/dL; cell counts in 10^3^/*μ*L. NS: not significant.

## Data Availability

The data used to support the findings of this study are included within the article.

## Additional Points

*Key Points*. Atherosclerotic coronary artery disease has long been considered a chronic low-grade subclinical systemic inflammatory disease. An independent association between the neutrophil-lymphocyte ratio and coronary atherosclerotic disease extent is implied in this study. The unique appreciation of this investigation is the simultaneous assessment of both coronary artery calcification and inflammation markers in asymptomatic and stable patients with clinical low-intermediate probability of coronary atherosclerotic disease.
